# Chronic Inflammation Links Cancer and Parkinson’s Disease

**DOI:** 10.3389/fnagi.2016.00126

**Published:** 2016-06-03

**Authors:** Zhiming Li, Zaozao Zheng, Jun Ruan, Zhi Li, Chi-Meng Tzeng

**Affiliations:** ^1^Translational Medicine Research Center, School of Pharmaceutical Sciences, Xiamen UniversityXiamen, China; ^2^Key Laboratory for Cancer T-Cell Theranostics and Clinical TranslationXiamen, China; ^3^INNOVA Cell TheranosticYangzhou, China; ^4^TRANSLA Health GroupXiamen, China

**Keywords:** PD, cancer, chronic inflammation, microglia, COX2, CARD15

## Abstract

An increasing number of genetic studies suggest that the pathogenesis of Parkinson’s disease (PD) and cancer share common genes, pathways, and mechanisms. Despite a disruption in a wide range of similar biological processes, the end result is very different: uncontrolled proliferation and early neurodegeneration. Thus, the links between the molecular mechanisms that cause PD and cancer remain to be elucidated. We propose that chronic inflammation in neurons and tumors contributes to a microenvironment that favors the accumulation of DNA mutations and facilitates disease formation. This article appraises the key role of microglia, establishes the genetic role of *COX2* and *CARD15* in PD and cancer, and discusses prevention and treatment with this new perspective in mind. We examine the evidence that chronic inflammation is an important link between cancer and PD.

## Introduction

The three early, typical symptoms of Parkinson’s disease (PD) are static tremors, muscle rigidity, and bradykinesia, which result from the degeneration of dopaminergic neurons in the midbrain. In contrast, cancer is a disease caused by the clonal proliferation of selectively advantageous cells. Although the two may appear distinct, epidemiological studies have revealed some parallels. Doshay (1954) found a decreased frequency of cancer incidence in patients with PD, but the reason for this was unknown. Case-controlled and large prospective studies reported a lower rate of smoking-related and non-smoking-related cancers in PD patients. Melanoma, thyroid, and breast cancer were revealed to have an increased incidence in PD patients (D’Amelio et al., 2009; Liu et al., 2011). There are a number of potential explanations for the inverse association. One of theories between PD and skin cancer may be associated with the mode of therapy, such as Levodopa, rather than with the disease itself (Paisan-Ruiz and Houlden, 2010). However, some observations do not agree with the view that this may be a result of drug treatment (Fiala et al., 2003; Zanetti et al., 2006). Moreover, it was suggested that PD patients with a low incidence of cancer may be associated with the negative correlation between PD and smoking (Hernan et al., 2002). Although the reduced risk of smoking-related cancers can be explained in PD patients, it cannot decipher the risk of non-smoking-related cancers. Thus, more data are needed to confirm the epidemiological relationship between PD and cancer.

**FIGURE 1:**
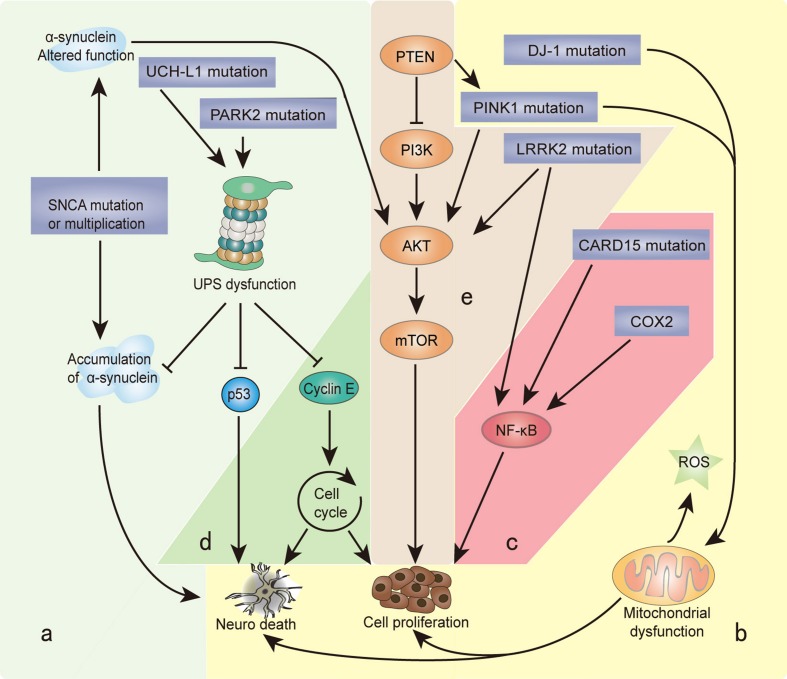
**Genes and biological processes shared in Parkinson’s disease (PD) and cancer.** Overlapping biological processes of PD and cancer mainly include the following: **(a)** misfolding and degradation of proteins, **(b)** mitochondrial damage and oxidative stress response, **(c)** chronic inflammation, **(d)** cell cycle control and DNA repair, and **(e)** PI3K/AKT/mTOR pathway regulation. The α-synuclein polymer attributed to *SNCA* multiplication is the main component of Lewy bodies (LBs). *SNCA* mutations alter the normal function of α-synuclein, which induces the PI3K/AKT/mTOR pathway and promotes cell proliferation. Under the cascade of phosphorylating AKT, mTOR is activated by PINK1 and LRRK2. *PARK2* and *UCH-L1* mutations disrupt the degradation function of the ubiquitin proteasome system (UPS), leading to the misfolding and aggregation of α-synuclein, cyclin E, and p53. *PINK1* and *DJ-1* mutations result in the overproduction of free radicals and oxidative stress in the mitochondria, which causes neuron defects and stimulates cell proliferation. *COX2* and *CARD15* mutations activate the NF-κB pathway and induce chronic inflammation, leading to a wide range of genetic mutations and abnormal cellular signaling. The different cellular backgrounds of cancer cells and neurons (mitotic vs. post-mitotic cells) bring completely distinct reactions to external stimuli and internal changes: some undergo cell proliferation, and others neuron death. The final result is two serious diseases: cancer and PD.

Recently, the unusual epidemiological information between PD and cancer is increasingly becoming of interest to many investigators. Genetic studies provide additional understanding, since many familial PD genes have been associated with cancer, such as *parkin* (*PARK2*), *PINK1* (*PARK6*), *DJ-1* (*PARK7*), and *LRRK2* (*PARK8*; **Table 1**). However, the same gene’s variants may lead to distinct effects due to the different cell backgrounds associated with dopaminergic neurons and cancer cells. PD-related genes participate in a wide variety of cellular processes, including the misfolding and degradation of proteins, mitochondrial damage, response to oxidative stress, cell cycle control, and DNA repair (**Figure 1**). These processes all have an important influence on the occurrence and development of PD and cancer. The PI3K/AKT/mTOR pathway also plays important roles in both cell growth and death. Some common cellular pathways and genes have been explained in our work (Zhiming et al., 2014). Notably, neuroinflammation is increasingly considered a double-edged sword (Wyss-Coray and Mucke, 2002). Acute inflammation can repair damage and promote healing, but long-term chronic inflammation can severely damage the body. Chronic inflammation is considered a driving force behind many chronic diseases, certainly including cancer and neurodegenerative disease. In this article, we further discuss the relationship between PD and cancer from the perspective of chronic inflammation. This will provide an increased understanding and improved treatment approaches for both diseases.

**Table 1:** Parkinson’s disease (PD) involved genes identified in cancer.

Gene	PD locus	Chromosome location	Inheritance in PD	Expression in cancer	Proliferation in cancer^b^	Cancer
*α-Synuclein*	*PARK1/PARK4*	4q21–q23	AD	Overexpressed (not express in normal tissue)	+	Brain tumors (Kawashima et al., 2000), Melanoma (Matsuo and Kamitani, 2010), and Ovary cancer (Bruening et al., 2000)
*Parkin*	*PARK2*	6q25.2–q27	AR	Decreased^a^	-	Glioblastoma, Colon cancer, and Lung cancer (Veeriah et al., 2010)
*UCHL1*	*PARK5*	4p14	AD	Silenced (via CpG methylation)	-	Nasopharyngeal carcinoma (Li et al., 2010), Colorectal cancer (Okochi-Takada et al., 2006)
*PINK1*	*PARK6*	1p35–p36	AR	Decreased^a^	-	Breast cancer (Berthier et al., 2011)
*DJ-1*	*PARK7*	1p36	AR	Overexpressed	+	Non-small-cell lung cancer (MacKeigan et al., 2003)
*LRRK2*	*PARK8*	12p11.2–q13.1	AD	Overexpressed	+	Papillary renal cell carcinoma and Thyroid cancer (Looyenga et al., 2011)


*AD, autosomal dominant; AR, autosomal recessive. ^a^The telomeric end of chromosome 1p is subject to frequent deletion and rearrangement in many cancers. ^b^± denotes proliferation and anti-proliferation.*

## Microglia: A Key Player

Inflammation and PD have an established link via microglia, which are the resident immune cells of the central nervous system (CNS). Under physiological conditions, microglia are inactive, have a small cell body, and exhibit highly ramified morphology. In response to injury or toxic factors, microglia transform into activated microglia, migrate to the lesion area, and phagocytose cellular debris or damaged neurons. It is now well documented that activated microglial surround the lost dopaminergic neurons in patients with PD and PD animal models, which provides for the speculation that microglia activation results in initiation and development of PD (Tansey and Goldberg, 2010). A recent investigation revealed that neurons could release α-synuclein oligomers, the main component of Lewy bodies (LBs) in the substantia nigra pars compacta (SNc) of the midbrain, which can bind to toll-like receptors (TLRs) to activate microglia, mediating the nuclear factor kappa B (NF-κB) pathway and causing the secretion of inflammatory mediators, such as cytokines, eicosanoids, and chemokines, altering immunological functions (Beraud and Maguire-Zeiss, 2012). These immune factors not only directly act on dopaminergic neurons to cause neuronal death but also aggravate the inflammatory reaction and continue to activate microglia. Activated microglia also generate and release a wide range of reactive-free radicals and increasing evidence has shown that the oxidative stress response plays a crucial role in PD. The sustained production of free radicals and noxious compounds, like superoxide or peroxynitrite, can interact with biological macromolecules, leading to disruption to the redox balance of neurons. Additionally, levels of pro-inflammatory mediators, including tumor necrosis factor (TNF)-α, interleukin (IL)-1β, IL-6, and eicosanoids are enhanced in peripheral blood mononuclear cells (PBMCs) of PD patients (Bessler et al., 1999). Recent studies have shown that inhibiting the microglia cascade reactions could prevent the degradation of neurons (Burguillos et al., 2011). The immunohistochemical demonstration of reactive microglia suggests that chronic inflammation occurs in affected brain regions in PD.

The brain is a common metastatic site for various types of cancers, especially lung cancer, which suggests failure of the immune defense in the brain environment. Microglia are believed to be the most important immune cells in the CNS. Interestingly, significant microglial activation has been observed in the vicinity of glioma tumor cells (Roggendorf et al., 1996). In contrast to CNS inflammation, microglia associated with brain tumors do not seem to be active in inducing an effective antitumor response. The exact mechanism of this microglial inactivation remains unclear. There is evidence that some cytokines, such as IL-10 released from activated microglia, play important roles in local immunosuppression and progression of glioma, particularly promoting proliferation of tumor cells and their infiltration into surrounding normal brain tissue (Wagner et al., 1999). Microglia also play an important role in phagocytosing tumor cells. During the past decades, more attention has been paid to the secretory property and chemotaxis of microglia, but microglial phagocytosis is not well-studied. The activity of phagocytosis can be modulated by cancer cells or the cancer environment. However, whether microglial phagocytosis functions to contribute to cancer or cancer defense still needs further investigation. A better understanding of microglia function is important for the development of immune-based treatment strategies against malignant brain tumors.

## Proven Genetic Risk Implicated in Both Cancer and PD

Inflammation is known as the seventh most important sign of cancer (Colotta et al., 2009). Sometimes, inflammatory mediators are referred to as genetic mutagens, which disturb DNA repair pathways and cell-cycle checkpoints, threatening the stability of the genome and accumulating chromosomal alterations. Inflammatory mediators in the microenvironment during chronic inflammation not only help cancer cells proliferate and escape from immune surveillance but also cause a large number of random mutations (Grivennikov et al., 2010). A major advantage of the cell cycle is careful checking and repair of the damaged DNA, but post-mitotic neurons are particularly vulnerable to the abnormal levels of mediators and accumulation of mutations. Some mutations in two genes unequivocally linked to chronic inflammation, namely *COX2* and *CARD15*, were identified in tissue samples from patients with PD and cancer.

### Cyclooxygenase 2 (COX2)

It is well known that prostanoids have critical functions in many cellular processes and pathophysiologic process, including inflammation, arthritis, and pain. COX-1 and -2 are two key enzymes in the synthesis of prostaglandins from of arachidonic acid. COX1 is constitutively expressed in the majority of tissues and is responsible for maintenance the normal physiological functions. Inversely, COX2 expression is low in most normal tissues, but it can be induced by various inflammatory stimuli (Smith et al., 2000). High levels of COX2 have been detected in many cancers, including gastric, breast, lung, esophageal, and hepatocellular carcinomas, particularly in colon cancer (Liu et al., 2003). Several lines of evidence suggest that non-steroidal anti-inflammatory drugs (NSAIDs) and selective COX2 inhibitors are promising as anti-cancer drugs. Genetic studies suggested that there is a strong causality between COX2 and tumorigenesis. Polymorphisms in the COX2 gene could alter enzyme expression, function, and/or the response to NSAIDs. The *COX2* V511A polymorphism was very near to the active site of a molecular model (Fritsche et al., 2001). In African Americans, the *COX2* V511A polymorphism was reported to confer a reduced susceptibility to colon cancer (Lin et al., 2002). COX2 has been shown to activate the NF-κB pathway and the p38 and JNK MAPK pathways (Hunot et al., 2004). Polymorphisms in the *COX2* and other genes these pathways may account for inter-individual differences in cancer susceptibility or response to NSAIDs.

Cyclooxygenase 2 is inducible by growth factors, cytokines, and pro-inflammatory molecules. COX2 expression in brain has been associated with pro-inflammatory activities. COX2 has been observed to increase in brain dopaminergic neurons of both PD post-mortem specimens and in the PD mouse model induced by 1-methyl-4-phenyl-1,2,3,6-tertrahydropyridine (MPTP). The involvement of COX2 in PD pathogenesis was further evidenced by the observation that MPTP neurodegeneration was alleviated in COX2, but not in COX1, knock out mice (Minghetti, 2004). Recently, the COX2 1195G > A polymorphism was considered to be a protective factor in the onset of PD in the Chinese Han population (Dai et al., 2015). However, the role of the COX2 variant on the generation of PD has rarely been studied. Previous studies found that different polymorphisms of some known genes related to PD may result in different diseases: PD or cancer due to the differentiated background of neuronal and cancer cells (Veeriah et al., 2010; Vasseur et al., 2012). Lopez de Maturana et al. (2014) found that LRRK2 also affected the inflammatory response in PD patients through regulating the expression of the COX2 enzyme. By extension, researchers propose that the overlap between PD and cancer is chronic inflammation, and COX2 may be the key enzyme in the inflammatory response to combine them.

### Caspase Recruitment Domain Protein 15 (CARD15)

Caspase recruitment domain protein 15, also known as nucleotide-binding oligomerization domain protein 2 (NOD2), is a protein that is encoded by the *CARD15* gene located on human chromosome 16q12 (Hampe et al., 2001; Hugot et al., 2001; Ogura et al., 2001). CARD15 is the receptor of muramyl peptides (MDP), a component of bacterial peptidoglycan. The binding of MDP and CARD15 activates NF-κB, resulting in inflammatory reactions. It was shown that *CARD15* mutations are involved in the innate immune system and pathogen recognition in terms of other complex polygenic diseases. In 2001, three laboratories identified genetic variants related to Crohn’s disease (CD), an inflammatory bowel disease. About 40% of CD patients in Western countries have at least one of the three SNPs: R702W, G908R, and L1007fsinsC. The heterozygous mutation of any of these SNPs increases the risk of CD by two- to fourfold. In addition, multiple-locus heterozygous mutations or homozygous mutations may lead to a more than 20-fold increased CD risk. Nevertheless, it was reported that none of these SNPs are involved in CD in Chinese (Han; Gao et al., 2005), Korean (Croucher et al., 2003), and Japanese patients (Yamazaki et al., 2002). Bialecka et al. (2007) showed that three SNPs in *CARD15* (R702W, G908R, and L1007fsinsC) were significantly related to PD patients in a Polish population. Our group identified P268S, an additional *CARD15* SNP that might be a risk factor for PD in a Chinese population (Ma et al., 2013). Additionally, Crane et al. (2002) reported that P268S was related to susceptibility of ankylosing spondylitis. Proell et al. (2008) performed sequence comparisons and found that CARD15 shared a high degree of similarity with apoptotic protease activating factor 1 (Apaf-1). They simulated the homologous structure of CARD15 based on the structure of Apaf-1 and found that P268S was located at the connexon (ligand-binding position) before the first helix of the NOD. Replacing Pro with Ser changed the conformation of the connexon and affected its binding to the substrate. These experimental observations show a possible linkage between the dysfunction of CARD15 and neurodegenerative disease.

Whether CD-related *CARD15* variants result in a loss or gain of effect of the CARD15 receptor is still unclear, and the mechanisms by which this alteration, in function, may enhance the susceptibility to CD need to be clarified. Patients with CD have an enhanced risk of developing colorectal cancer (Gasche and Carethers, 2004; D’Amelio et al., 2009). *CARD15* mutations may also increase the susceptibility to colorectal cancer in non-CD Caucasians (Papaconstantinou et al., 2005; Roberts et al., 2006). These investigations demonstrated that immune system mechanisms are involved in the etiology of cell damage in CD and provided evidence for an ongoing active pathological process. Inflammation can be triggered by invading microbes and also internally within the organism by diseases that affect the nervous system. There are three common outcomes of inflammation. First, the offending agent or process is inactivated, and the injury is repaired. Second, the host loses the battle and dies or suffers irreparable tissue damage. Finally, neither the organism nor the injurious process prevails, resulting in a prolonged battle that provides a fertile ground for the development of chronic inflammatory conditions. This last outcome may be closely related to neurodegenerative diseases and cancer, two of the greatest public health problems of this century (Wyss-Coray and Mucke, 2002).

## Therapeutic Interventions Inspired By Their Links

Lower rates of cancer mortality and incidence in patients with PD have given rise to speculation about risks or preventative factors common to both diseases. Thus, unraveling the link between PD and cancer may open a new therapeutic window for both diseases. Epidemiological investigations revealed that taking NSAIDs can help lower your risk of PD development, and patients with colorectal cancer treated with NSAIDs showed 40–50% decreased mortality compared with those not using these drugs (Subbaramaiah and Dannenberg, 2003). The important role of mitotic activation in neurodegeneration raises the possibility of the cell cycle as a therapeutic target. Drugs, such as retinoic acid, simvastatin, and IL-1, which lead to cell cycle arrest at the G0/G1 interface, might prevent neurons from committing themselves to mitosis and therefore apoptosis. Another intervention would be to interfere with cellular signaling cascades that drive cell cycle progression or apoptosis, like Mithramycin (Sleiman et al., 2011). A number of other compounds have anti-neoplastic and neuroprotective properties, including inhibitors of histone deacetylase (Sleiman et al., 2009), mTOR (mammalian target of rapamycin), and transglutaminase (Ravikumar et al., 2004). Along with the cell cycle, the ubiquitin proteasome system is a common link between cancer and neurodegeneration, and an exciting target for new therapies. Because defective proteolysis is common to all neurodegenerative diseases, the potential clinical effect of a proteasome activator is beneficial and should be a focus of drug development. Trials of proteasome activators in PD should be carefully designed to capture any result of a related increased risk for malignancy because upregulation of the proteasome is associated with many cancers.

In 2010, Datamonitor Inc., (USA) estimated that there were over 1.5 million PD patients in the United States, Japan, France, Germany, Italy, Spain, and UK combined, one third of which are in the United States. Combined with the aggravated aging problem, the incidence of PD is increasing annually (Huynh, 2011). Medication is usually the first treatment option for PD. Levodopa is currently the most effective medication, but long-term use can reduce its effectiveness and cause complications such as motor dysfunction. Most brain degenerative diseases are incurable, and it is difficult to obtain the brain tissues from the patients with significant impairment. However, large amounts of cancer-related studies have been carried out over the past 30 decades, and many small molecule agents have been demonstrated as cancer therapeutic drugs or are now being assessed in clinical trials. Therefore, the extensive therapeutic developments in cancer studies may help the identification of diagnostic and prognostic markers for neurodegeneration that could contribute to improve treatments for PD.

## Conclusion

Both cancer and PD are considered to be the consequence of the interaction of genes and environmental factors. The key difference is that different reactions occur in the different cellular backgrounds of cell division and cell death. The inflammation hypothesis is one explanation for comparisons between PD and cancer. The immune factors and free radicals released from chronic inflammatory reactions not only promote disease occurrence but also allow cellular DNA to accumulate mutations more easily, forming proteins with aberrant functions. The abundant therapeutic achievements in cancer studies will allow researchers to identify diagnostic markers and treatments for neurodegenerative disease and *vice versa*.

## Author Contributions

ZZ and JR designed the figure. Zhi Li designed the table. ZL and C-MT drafted the manuscript.

## Conflict of Interest Statement

The authors declare that the research was conducted in the absence of any commercial or financial relationships that could be construed as a potential conflict of interest.
